# Surface carboxylation or PEGylation decreases CuO nanoparticles’ cytotoxicity to human cells in vitro without compromising their antibacterial properties

**DOI:** 10.1007/s00204-020-02720-7

**Published:** 2020-04-07

**Authors:** Anna-Liisa Kubo, Grigory Vasiliev, Heiki Vija, Jekaterina Krishtal, Vello Tõugu, Meeri Visnapuu, Vambola Kisand, Anne Kahru, Olesja M. Bondarenko

**Affiliations:** 1 Laboratory of Environmental Toxicology, National Institute of Chemical Physics and Biophysics, Akadeemia tee 23, Tallinn, Estonia; 2 Department of Chemistry and Biotechnology, School of Science, TalTech, Akadeemia tee 15, Tallinn, Estonia; 3 Institute of Physics, University of Tartu, W. Ostwaldi 1, Tartu, Estonia; 4 Estonian Academy of Sciences, Kohtu 6, Tallinn, Estonia

**Keywords:** Surface coating, Nanosafety, Nanomedicine, Antibacterial, Immunotoxicity, Particle internalization

## Abstract

Clinical use of CuO nanoparticles (NPs) as antibacterials can be hampered by their toxicity to human cells. We hypothesized that certain surface functionalizations of CuO NPs may render NPs toxic to bacteria, but still be relatively harmless to human cells. To control this hypothesis, the toxicity of differently functionalized CuO NPs to bacteria *Escherichia coli* vs human cells (THP-1 macrophages and HACAT keratinocytes) was compared using similar conditions and end points. CuO NPs functionalized with polyethylene glycol (CuO–PEG), carboxyl (CuO–COOH, anionic), ammonium (CuO–NH_4_^+^, cationic) and unfunctionalized CuO NPs and CuSO_4_ (controls) were tested. In general, the toxicity of Cu compounds decreased in the following order: CuO–NH_4_^+^ > unfunctionalized CuO > CuSO_4_ > CuO–COOH > CuO–PEG. Positively charged unfunctionalized CuO and especially CuO–NH_4_^+^ proved most toxic (24-h EC_50_ = 21.7–47 mg/l) and had comparable toxicity to bacterial and mammalian cells. The multivariate analysis revealed that toxicity of these NPs was mostly attributed to their positive zeta potential, small hydrodynamic size, high Cu dissolution, and induction of reactive oxygen species (ROS) and TNF-α. In contrast, CuO–COOH and CuO–PEG NPs had lower toxicity to human cells compared to bacteria despite efficient uptake of these NPs by human cells. In addition, these NPs did not induce TNF-α and ROS. Thus, by varying the NP functionalization and Cu form (soluble salt vs NPs), it was possible to “target” the toxicity of Cu compounds, whereas carboxylation and PEGylation rendered CuO NPs that were more toxic to bacteria than to human cells envisaging their use in medical antibacterial products.

**Electronic supplementary material:**

The online version of this article (10.1007/s00204-020-02720-7) contains supplementary material, which is available to authorized users.

## Introduction

Increasing resistance of bacteria to conventional antibiotics necessitates the development of alternatives such as silver and copper-based antimicrobials, including in nanoformulations. Copper is known since long time as a metal with antibacterial effect that can be used to inhibit bacterial spreads by employing Cu on surfaces (Rosenberg et al. 2018), in aqueous suspension (Bastos et al. 2018) and in textiles (Teli and Sheikh 2013; Mantecca et al. 2017). For living organisms, including humans, Cu is an essential microelement. Cu is vital for, e.g., functioning of the innate and adaptive immune system (Percival 1995, 1998) and is the necessary component of the key enzymes (O’Dell 1976). Previous studies have shown that CuO NPs support wound healing (Borkow et al. 2010) and bone regeneration (Shi et al. 2016). For instance, mesoporous silica NPs containing 2.5–5% Cu were suggested for the use in bone regeneration, since they up-regulated the genes contributing to osteogenic and angiogenic factors and were not toxic in the range of 10–500 mg/l (i.e., 0.5–25 mg Cu/l) to murine macrophages RAW 264.7, whereas Cu significantly contributed to the beneficial properties of these NPs (Shi et al. 2016).

Given the above-mentioned properties, CuO NPs are ideal candidates for the use in medicine as wound dressings and/or internal implants by combining two functions: antimicrobial activity and increased wound healing or osteogenesis. However, the excessive copper is toxic and plays a role in the pathogenesis of several diseases (Klaassen and Curtis 2008; Brewer 2010; Montes et al. 2014). In case of topical use (e.g., in wound dressings), CuO NPs will be in close contact with keratinocytes and in case of internal use (e.g., implants), with the macrophages residing in the blood and tissues. Thus, it is important to avoid toxicity of CuO NPs to these cell types. Previous studies have shown that pristine (unfunctionalized) CuO NPs were toxic to murine macrophage cell line RAW264.7 (Líbalová et al. 2018) and other human cell models in vitro such as epidermal keratinocytes NHEK (Murugan et al. 2017), lung adenocarcinoma cells A549 (Karlsson et al. 2008), hepatoma cell line HepG2 (Piret et al. 2017), epithelial colon carcinoma cells Caco-2 (Käkinen et al. 2016) and differentiated Caco-2 (in vitro model for the cells of small intestine) (Ude et al. 2017) with the range of EC_50_ values of 13–100 mg/l (Bondarenko et al. 2013). However, the antibacterial concentrations of CuO NPs were in the range of 20–280 mg/l, implying that the therapeutic use of the existing (mostly unfunctionalized) CuO NPs as antibacterials is rather limited, since the CuO NPs effective in killing bacteria were also toxic to human cells in vitro (Bondarenko et al. 2013, 2016). Thus, successful commercialization of antibacterial CuO NPs necessitates a compromise of reasonable antibacterial properties with reasonable safety to human cells. As the prerequisite of the toxic action of chemicals/NPs is adversely influencing or crossing the main biological barrier (the cell wall/membrane that is different in bacterial and mammalian cells), we hypothesized that certain type of surface functionalization of CuO NPs may render NPs toxic to bacteria, but still be relatively benign to human cells. Indeed, surface functionalization of metal-based NPs may change the safety profile of NPs (Nel et al. 2009; Kubo et al. 2018). For example, functionalization of NPs with PEG, chitosan or dextran prevented the opsonization of NPs (i.e., adsorption of bio-corona of proteins and other biomolecules onto their surface) and, thus, reduced the uptake of NPs by macrophages and, hence, toxicity (Sheng et al. 2009; Jenkins et al. 2016; Wonder et al. 2018). In contrast, compared with NPs functionalized with neutrally (e.g., with PVP) or negatively charged coatings (e.g., citrate), functionalization of NPs with positively charged groups such as polyethylenimine (PEI), branched PEI or amine group rendered NPs that were more toxic to mammalian cells including murine macrophage cell line RAW264.7 (Líbalová et al. 2018), epithelial cells BEAS-2B and human monocytes THP-1 (Li et al. 2014).

While there are many articles on the biological effects of unfunctionalized CuO, the information on differently functionalized CuO NPs is rare (Bondarenko et al. 2013; Juganson et al. 2015). Although there are various protocols available for the synthesis of CuO NPs functionalized with, e.g., peptides, antibodies and oligonucleotides (Tauran et al. 2013), these NPs were mostly intended for bioanalytical applications and thus not tested for their potential toxic effects. Our search in PubMed (performed in December 2019) using the keywords “copper nano* tox*” identified in total 215 research articles, and only 8 of these addressed the biological effects of differently functionalized CuO NPs with the focus on the “green” functionalization such as chitosan (Worthington et al. 2013; Vanti et al. 2019), plant latex (Valodkar et al. 2011), albumin (Azizi et al. 2017) and a set of coatings including citrate, sodium ascorbate, polyvinylpyrrolidone, polyethylenimine (Líbalová et al. 2018) and, similarly to this study, carboxyl, PEG and ammonium (Meissner et al. 2019; Ilves et al. 2019). None of the studies compared the antibacterial properties of NPs with their safety to human cells in vitro or in vivo.

This study is the first report on the comparison of the antimicrobial efficiency and safety toward human cells of CuO NPs with different surface functionalizations: CuO–NH_4_^+^, CuO–COOH, CuO–PEG and unfunctionalized CuO NPs as well as CuSO_4_ as an ionic control. THP-1-derived macrophages were used as a model for immunotoxicity, HACAT keratinocytes in vitro as the model for human skin cells and *Escherichia coli* as model bacteria. We chose Gram-negative bacterium *E. coli* as there is a warning rise of multidrug resistance in Gram-negative bacteria becoming a challenge in health care (Exner et al. 2017). To minimize the effects of speciation of copper on test results, the toxicity of Cu compounds to THP-1 cells and bacteria was tested in comparable conditions using RPMI medium supplemented with 10% fetal bovine serum and 24-h Alamar Blue to determine cell viability. In addition, we compared the potential mechanisms of toxicity of studied Cu compounds to different cell types with the focus on reactive oxygen species (ROS), dissolution, cellular internalization of CuO and their ability to induce inflammation in mammalian cells, and revealed the main parameters contributing to toxicity using statistical multivariate analysis.

## Materials and methods

The manuscript does not contain clinical studies or patient data.

### Chemicals

All the purchased chemicals were at least of analytical grade. Dulbecco’s phosphate-buffered saline (DPBS, Biognost), Alamar Blue (AppliChem), CuSO_4_ (Alfa Aesar), 2′,7′-dichlorodihydrofluorescein diacetate (H_2_DCF-DA, Life Technologies), phosphate buffered saline (PBS pH = 7.2, Biognost), tryptone (LabM), yeast extract (LabM), agar (LabM) and NaCl (Sigma-Aldrich) were used.

### Nanoparticles

Four types of differently functionalized and unfunctionalized CuO NPs were obtained via the consortium of EU FP7 project NANOSOLUTIONS (https://nanosolutionsfp7.com/) as a kind gift from Prof. Bengt Fadeel (Karolinska Institutet, Sweden). CuO NPs were synthesized by PlasmaChem (Germany) by decomposition of Cu_2_CO_3_(OH)_2_, followed by the introduction of the surface groups via treatment with mercaptopropionic acid. CuO NPs were provided as dry powders, and the suspensions were prepared each time freshly before the tests at concentrations 1000–2000 mg compound/l in endotoxin free bi-distilled water (DI water). Ten milliliters of CuO NP suspensions were vortexed and sonicated using probe sonication (Branson 450 Sonifier, USA) for 5 min with acoustic power of 13 W corresponding to the specific energy of 3.9·10^5^ kJ/m^3^ (Käkinen et al. 2016).

The morphology and primary size of NPs were studied using transmission electron microscope (TEM) Tecnai G2 Spirit BioTwin (FEI) at 120 kV. A drop of a 200 mg/l NP suspension in methanol was deposited onto 200 mesh formvar/carbon coated copper grid (Agar Scientific, UK). Sixty particles were measured from TEM images using ImageJ software to obtain nanoparticle primary size. TEM figure for CuO-PEG was provided by NANOSOLUTIONS consortium (Fig. S1d).

Fourier transform infrared spectroscopy (FTIR) spectra were measured in the 1000–4000 cm^−1^ range with 2 cm^−1^ resolution using Bruker VERTEX 70 spectrometer with an attenuated total reflection (ATR) accessory.

Hydrodynamic size (Dh), polydispersity index (pdi) and zeta potential (Z-potential) of NPs were measured in 100 mg/l suspensions in DI water or cell culture medium using Malvern zetasizer (Zetasizer Nano-ZS, Malvern Instruments, UK).

The endotoxin content in CuO dispersions was assessed using the chromogenic Limulus amebocyte lysate (LAL) assay (Charles River Endosafe, Charleston, SC) according to the manufacturer’s instructions and was below the detection limit of the assay.

The Cu content of the tested Cu compounds was determined using total reflection X-ray fluorescence (TXRF, Picofox S2, Bruker Corporation) from 100 mg/l suspensions. Briefly, 40 µl of the sample was mixed with 40 μl of the reference element (2 mg/l Ga) and 3 μl of the mixture was pipetted onto quarts sample holder (Analyslide Petri Dish, Pall Corporation). The measurements were done in triplicate in at least two independent experiments.

For the dissolution analysis, 100 mg/l CuO NPs or CuSO_4_ (a recovery control) was incubated in cell culture medium (at 37 °C, 5% CO_2_ and 95% humidity) for 0 h, 30 min or 24 h and centrifuged at 320,000×*g* for 30 min (Bekman Coulter ultracentrifuge). After centrifugation, the supernatants were collected and analyzed by TXRF as described above.

### Human cell lines

The cell lines were obtained from American Type Culture Collection (ATCC) and cultured according to ATCC guidelines. The cells were subcultured up to 20 passages, and the toxicity tests were performed after at least two passages.

The human monocytic leukemia cell line THP-1 (ATCC TIB-202) was grown in Roswell Park Memorial Institute medium with l-glutamine (RPMI-1640, Corning) supplemented with 10% fetal bovine serum (FBS, Corning), 100 mM sodium pyruvate solution (Na-Pyr, Gibco) and 10,000 U/ml penicillin and 10,000 µg/ml streptomycin (PEST, Gibco) that is further referred to as the complete cell culture medium (CCM). THP-1 cells (growing in suspension) were subcultured by adding fresh CCM. Before the assays, THP-1 cells were differentiated into macrophage like cells by culturing them with 100 ng/ml phorbol myristate acetate (PMA, InvivoGen) in CCM. For that, THP-1 cells were seeded into 96-well plates (Corning Falcon) at a density of 10^5^ cells per well and incubated with 100 ng/ml phorbol myristate acetate (PMA) for 3 days at 37 °C and 5% CO_2_.

The human HACAT cell line, immortalized keratinocytes (ATCC PCS-200-011), were grown in Dulbecco`s modified Eagle’s medium with 4.5 g/l glycose, l-glutamine and sodium pyruvate (DMEM, Corning) supplemented with 10% FBS and 1% PEST. Before the tests, cells were seeded into 96-well plates at a density of 10^4^ cells per well and incubated for 1 day at 37 °C, 5% CO_2_ and 95% humidity. The composition of the test media used is shown in Table S1.

### Bacterial cells

*Escherichia coli* MG1655 (obtained from the *E. coli* genetic stock center, Yale University) and recombinant bioluminescent *E. coli* MC1061 (pSLcueR/pDNPcopAlux) [constructed in our laboratory previously (Ivask et al. 2009)] were stored on agarized Luria–Bertani medium (LB, 1% tryptone, 0.5% yeast extract, 0.5% NaCl, 1.5% agar) and before the toxicity tests cultivated in 3 ml of LB medium at 37 °C with shaking at 200 rpm overnight. In case of recombinant bacteria, LB was supplemented with 100 µg/l ampicillin and 10 µg/l tetracycline to retain the bioluminescence-encoding plasmid.

### Toxicity assays

**Fig. 1:**
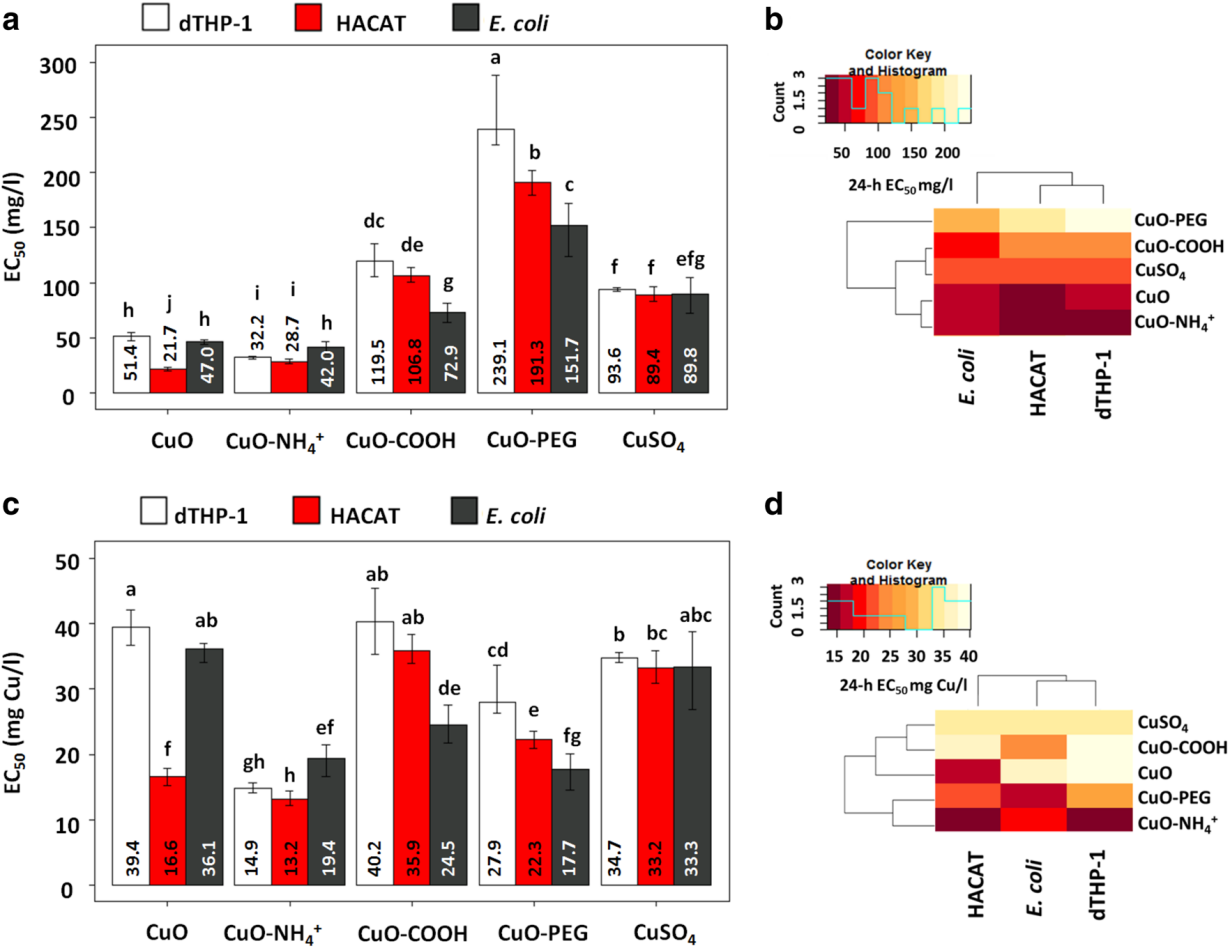
Toxicity of Cu compounds to bacteria *Escherichia coli* (*E. coli*), HACAT keratinocytes and differentiated THP-1 cells (dTHP-1). The average compound-based 24-h EC_50_ values with 95% confidence intervals mg/l (**a**) and the clustering of average compound-based 24-h EC_50_ (**b**). The average copper-adjusted 24-h EC_50_ values with 95% confidence intervals mg/l (**c**) and the clustering of average copper-adjusted 24-h EC_50_ (**d**). Data presented as bars with the same letters are not statistically significant, whereas data presented as bars with different letters are statistically significant

The toxicity of Cu compounds to *E. coli* and THP-1 cells was assessed in similar conditions (24-h incubation in CCM medium at 37 °C and using Alamar Blue assay for viability evaluation) with minor differences: (1) PEST was removed from *E. coli* exposure medium; (2) human cells were incubated in humidified conditions (5% CO_2_). Details on the test conditions are summarized in Table S1. All reported concentrations were nominal and EC_50_ values were calculated either based on the compound (compound-based concentrations, Figs. 1a and S3, left panel) or on copper (copper-adjusted concentrations, Figs. 1c and S3, right panel) to estimate the contribution of Cu to the toxicity. The concentration of copper in Cu compounds was determined by TXRF as described above.

*E. coli* cells were grown in LB medium overnight, followed by removal of the medium by centrifugation and resuspension of bacterial pellet in CCM without PEST to ~ 5 × 10^5^ colony forming units (CFU/ml). For the toxicity assay, 100 μl of bacterial suspension was exposed to 100 μl of either cell culture medium (control) or 6.25–400 mg/l CuO suspensions/CuSO_4_ in CCM in transparent 96-well plates for 24 h at 37 °C. The bacterial viability was estimated using Alamar Blue assay. For that, the exposed cells were washed and Alamar Blue (AppliChem, final concentration of 150 μg/ml) in CCM without PEST was added to the cells for 2 h at 37 °C. After incubation, fluorescence was read by Fluoroscan (Fluoroskan Ascent FL, Thermo Labsystems) with excitation at 530 nm and emission at 590 nm. The metabolic activity (viability) of the exposed cells was expressed in % by comparing their fluorescence with that of untreated cells. The EC_50_ values were calculated as described in ”Statistical analysis”. Tests were performed in five biological experiments in duplicate. To assess possible interference of NPs with assay reagents, NPs with Alamar Blue were also incubated in abiotic conditions (no unspecific reactions were observed).

For the toxicity assay with human cells, the cell culture medium was removed, cells were washed with PBS and exposed to 100 μl of either cell culture medium or Cu compounds in cell culture medium for 24 h at 37 °C and 5% CO_2_. After the exposure, the supernatant was removed, cells were washed once with PBS and incubated with 100 µl of 150 µg/ml Alamar Blue for 2 h at 37 °C and with 5% CO_2_.

### Bioavailability of Cu to bacteria

Quantification of intracellular Cu ions was performed using recombinant biosensor bacteria *E. coli* MC1061 (pSLcueR/pDNPcopAlux) in which Cu ion-inducible promoter *copA* is genetically coupled to the bioluminescence-encoding genes *luxCDABE* (Ivask et al. 2009). Thus, bioluminescence of this recombinant *E. coli* increases in response to sub-toxic concentrations of intracellular Cu ions in a dose-dependent manner. In the toxic concentration range, the bioluminescence of bacteria gradually decreases.

The overnight bacterial culture was diluted 1:20 into fresh LB medium supplemented with 100 µg/l ampicillin and 10 µg/l tetracycline, grown till OD = 0.5–0.8 and diluted in CCM without PEST to OD = 0.1 corresponding to a final concentration of 10^6^ CFU/ml. 100 μl of the appropriate dilution of Cu compounds in CCM without PEST was pipetted into the wells of white 96-well microplates and 100 μl of bacterial culture in CCM without PEST was added. The test plates were incubated at 37 °C for 2 h, and bioluminescence was measured using Orion II plate luminometer (Berthold Detection Systems). Fold increase in bioluminescence in response to Cu compounds was calculated as a function of increased bioluminescence of biosensor in the sample (CuO and CuSO_4_ dilutions in CCM without PEST) compared to the background (CCM without PEST).

### Measurement of reactive oxygen species

The ability of CuO NPs and CuSO_4_ to generate ROS was measured in abiotic conditions in DI water with H_2_DCFA-DA as described by Aruoja et al. (2015). 100 μl of 6.25–200 mg/l CuO NPs and CuSO_4_ and 100 μl of H_2_DCF were incubated at room temperature (RT) for 60 min. Fluorescence (excitation at 485 nm and emission at 527 nm) was quantified using a microplate fluorometer (Fluoroskan Ascent FL, Thermo Labsystems, Finland). The ability of Cu compounds to induce ROS was expressed in % in relation to the control.

### Chemical analysis of cell-associated Cu

THP-1 monocytes were seeded into 96-well plates (Corning Falcon) at a density of 10^5^ cells/well and differentiated with 100 ng/ml PMA for 72 h. Cells were exposed to Cu compounds in CCM at EC_20_ concentrations for 24 h (27.3 mg/l for CuO NPs, 22.2 mg/l for CuO-NH_4_^+^, 90.6 mg/l for CuO-COOH, 211.4 mg/l for CuO-PEG and 85.4 mg/l for CuSO_4_).

HACAT cells were seeded into 96-well plates at density 10^4^ cells/well and allowed to attach for 24 h. The cells were exposed to Cu compounds at EC_20_ concentrations (11.6 mg/l for CuO NPs, 14.9 mg/l for CuO–NH_4_^+^, 73.7 mg/l for CuO–COOH, 142.0 mg/l for CuO–PEG and 57.6 mg/l for CuSO_4_) for 24 h.

After 24 h exposure, the cells were washed, detached and washed again twice with PBS by centrifugation at 150×*g* for 5 min. 10 μl cell suspension was mixed with 10 μl trypan blue and the cell number and cell viability were determined. The supernatant was aspirated and the pellet was lyophilized. The Cu content was quantified with TXRF, normalized on total cell number basis and designated as “cell-associated Cu”, referring to the sum of the following fractions: intracellular Cu and extracellular Cu bound to the cell surface.

### Measurement of TNF-α

Differentiated THP-1 cells at density 10^5^ cells/well were exposed to CuO NPs and CuSO_4_ at concentrations from 25 to 400 mg/l in CCM. After 24-h exposure, the supernatants were collected, centrifuged for 10 min at 10,000×*g* and stored frozen at – 80 °C. TNF-α was measured on 96-well plates using Enzyme-Linked Immunosorbent Assay (ELISA) kit (Invitrogen 88-7346) according to the manufacturer’s instructions.

### Microscopy

For the automatic photographing, THP-1 cells were differentiated in 24-well plates, exposed to NPs (24-h EC_20_ concentrations), washed, stained with Giemsa Stain (Sigma-Aldrich) according to manufacturer’s instructions and visualized using Automated Digital Morphology System CellaVision^®^. Before the analysis, differentiated THP-1 cells were mixed with human red blood cells to improve the cell recognition by the software.

For the confocal microscopy, THP-1 cells were differentiated on glass coverslips in 12-well plates, stained with 5 µg/ml Cell Mask Orange (CMO) cell membrane dye (Invitrogen), fixed with 4% paraformaldehyde (Sigma) and stained with 1:300 diluted DAPI (Sigma). Finally, the coverslips were rinsed and mounted with ProLong^®^ Gold antifade reagent (Life Technologies) for 12–24 h at room temperature in the dark. Cells and NPs were visualized using a confocal microscope Zeiss Duo 510 META with 63× oil immersion objective 1.4 NA. To set up the reflectance optical configuration, the main beam splitter was set to NT80/20 and the channel was set up for reflectance using the 488 nm laser. CMO was excited with 561 nm laser and DAPI was visualized with 405 nm laser. Z-stacks from the coverslip to the top of the cell were acquired at a step size of 320 nm. For three-dimensional (3D) reconstruction Imaris 6.4.2 software was used.

### Statistical analysis

All tests were performed in at least three individual experiments in duplicate. The EC_50_ values were calculated using MS Excel macro Regtox (https://www.normalesup.org/~vindimian/en_download.html) and the results were presented with 95% confidence intervals. The statistical significance between the EC_50_ values was estimated assuming equal variances at *p* < 0.05 with one-way ANOVA followed by Tukey`s HSD post hoc test. Heatmap and dendrogram were done with R Language and Environment for Statistical Computing (https://www.R-project.org). Heatmaps and dendrograms were generated using heatmap function (incorporating Euclidean distance and complete method).

Principal component analysis (PCA) was used to obtain a multiparametric estimation of the variables that contributed to the toxicity (average compound-based EC_50_ values) of CuO NPs. Scores of the first two PCs which accounted for 87–95% of the variance were used to generate the biplots*.* For visualization, data were scaled by dividing the (centered) columns of *x* by their standard deviations.

## Results

### Physico-chemical characterization of CuO NPs

The primary sizes of CuO NPs were measured by transmission electron microscopy (TEM, Fig. S1) and the presence of the different organic functional groups on the functionalized NPs was verified with Fourier transform infrared spectroscopy (FTIR) (Fig. S2). CuO NPs mostly formed agglomerates of a few hundred nanometers with the primary particle sizes of 15.9 ± 5.2, 6.9 ± 2.2, 9.2 ± 2.5 and 12.1 ± 3.2 nm for the CuO, CuO–COOH, CuO–NH_4_^+^ and CuO–PEG NPs, respectively (Fig. S1; Table 1).

**Table 1:** Physico-chemical characteristics of Cu compounds

Cu compounds	Primary size, nm^a^	Hydrodynamic diameter (Dh) in DI water^b^ nm (pdi^c^)	Dh in cell culture medium^b^, nm (pdi)	Z-potential in DI water^b^, mV	Z-potential in cell culture medium^b^, mV	Cu content^d^, %
CuO NPs	15.9 ± 5.2	237 ± 31 (0.25)	204 ± 13 (0.45)	27.5 ± 1.8	− 10.8 ± 1.4	76.8 ± 5.7
CuO−NH_4_^+^ NPs	6.9 ± 2.2	733 ± 252 (0.24)	936 ± 229 (0.67)	25.8 ± 1.3	− 8.9 ± 0.8	46.2 ± 4.0
CuO−COOH NPs	9.2 ± 2.5	1124 ± 128 (0.35)	303 ± 84 (0.70)	− 12.0 ± 2.2	− 10.2 ± 0.8	33.6 ± 3.2
CuO−PEG NPs	12.1 ± 3.2	1244 ± 254 (0.35)	1268 ± 315 (0.88)	− 21.9 ± 3.3	− 10.0 ± 1.8	11.7 ± 1.0
CuSO_4_	NA	NA	NA	NA	NA	37.1 ± 4.5

*NA* not applicable

^a^Measured by transmission electron microscopy (TEM)

^b^Measured by Malvern Zetasizer from 100 mg/l suspensions

^c^Polydispersity index

^d^Analyzed by TXRF from 100 mg/l suspensions

FTIR spectra proved the presence of organic functional groups as absorption peaks characteristic to O–H, C–H, C=O, N–H and C–O vibrational bands were identified in the measured spectra (Fig. S2).

Hydrodynamic size (Dh) of NPs was in the range of 204 nm (CuO NPs) to 1268 nm (CuO-PEG) (Table 1). The polydispersity index (pdi) values did not exceed 0.35 in the DI and increased to 0.45–0.88 in the cell culture medium, confirming the tendency of NPs, especially CuO–PEG (pdi = 0.88), to agglomerate in the test medium. The Z-potential reflecting the particle surface charge in DI water was positive for CuO and CuO–NH_4_^+^ and negative for CuO–COOH and CuO–PEG. In the cell culture medium, the Z-potential of NPs was negative for all the particles ranging from – 8.9 mV (CuO–NH_4_^+^) to – 10.8 mV (CuO), most likely due to the adsorption of the serum proteins (the Z-potential of the test medium alone was – 10.4 mV) as suggested previously by Ivask et al. (2015) or the interference of the serum proteins [such as negatively charged bovine serum albumin tending to adsorb to the particles (Jachimska and Pajor 2012)] with the measurement. Measured total Cu content was the highest for CuO (76.8%), followed by CuO–NH_4_^+^ (46.2%), CuO–COOH (33.6%) and CuO–PEG (11.7%). The measured total Cu content in CuSO_4_ was 37.1 ± 4.5%, in agreement with the calculated amount of Cu in CuSO_4_ (39.8%), and Cu content in CuO was 76.8 ± 5.7% close to the calculated amount of Cu in CuO (79.9%) (Table 1).

### Toxicity of Cu compounds

The loss of viability of the cells after 24-h exposure to different copper compounds is shown in Fig. S3. Figure 1 depicts the average 24-h EC_50_ values calculated on the basis of the dose–response curves from Fig. S3 (left panel) and dendrogram showing the clustering of these EC_50_ values (Fig. 1b). While the 24-h EC_50_ values of CuSO_4_ were very similar for all cell types, the toxicity of NPs to different cells significantly varied (Fig. 1a). Namely, unfunctionalized CuO and CuO–NH_4_^+^ were more toxic to human cells in vitro than to bacteria, whereas negatively charged NPs—CuO–COOH and CuO–PEG—were significantly more toxic to bacteria compared to human cells. Thus, clearly, by varying the NP surface functionalization and also Cu form (soluble salt vs NPs), it was possible to tune the toxicity of Cu compounds to bacteria vs to human cells.

Dendrogram analysis of the average 24-h EC_50_ values pointed out several clusters: most toxic NPs—unfunctionalized CuO and CuO−NH_4_^+^—clustered together, whereas CuO and CuSO_4_ formed another cluster and the least toxic CuO-PEG NPs a separate cluster (Fig. 1b). Since the most toxic NPs (unfunctionalized CuO and CuO−NH_4_^+^ NPs) contained the highest % of Cu (Table 1), the 24-h EC_50_ values of Cu compounds were re-calculated based on Cu content (from Table 1) and presented in Fig. 1c. Cu-adjusted EC_50_ of CuSO_4_ proved to be around 33 mg Cu/l for all cell types. Cu-adjusted EC_50_ values of CuO NPs were mostly lower or the same as for CuSO_4_ depending on the surface functionalization and cell type showing that not only Cu contributed to the toxicity. Interestingly, Cu-adjusted EC_50_ values clustered differently, highlighting that CuO−NH_4_^+^, CuO−PEG and CuO−COOH NPs are the most potent antibacterials (Fig. 1d). While CuO−PEG and especially CuO−COOH NPs were less toxic to human cells compared to bacteria, CuO−NH_4_^+^ NPs were more toxic to mammalian cells. In addition, the Cu-based EC_50_ values of CuO−NH_4_^+^ NPs were about twice lower than that of CuSO_4_ (Fig. 1c), suggesting that the toxicity of CuO−NH_4_^+^ NPs cannot be solely explained by Cu content and additional toxicity mechanisms played a role in its toxicity. Thus, we determined the ability of Cu compounds to induce ROS in abiotic conditions and inflammation in mammalian cells and studied in detail their interactions with bacterial and human cells in vitro with the focus on NP localization and uptake mechanisms.

### Mechanisms of toxicity of Cu compounds

#### Bioavailability and dissolution of Cu compounds

**Fig. 2:**
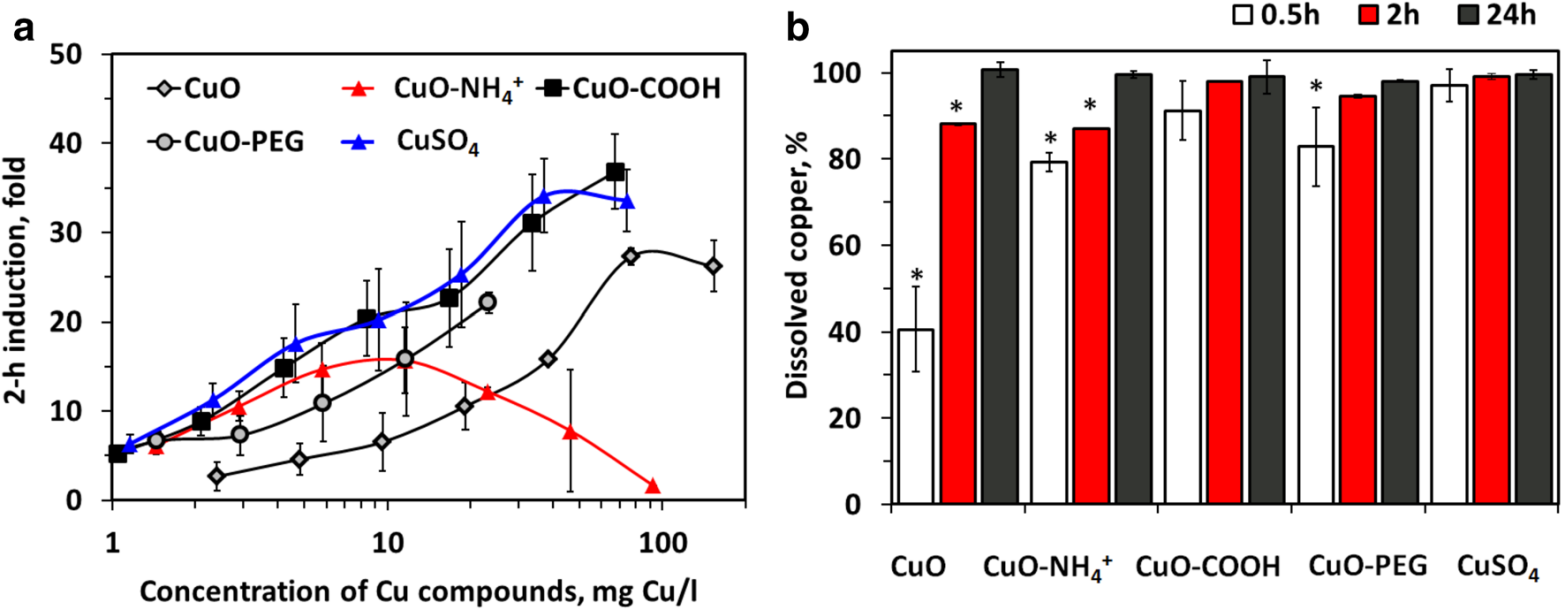
Bioavailability and dissolution of Cu compounds. Induction of bioluminescence in *E. coli* biosensor in response to Cu compounds (**a**) and abiotic dissolution of Cu compounds in cell culture medium (100 mg/l, 37 °C) after 0.5, 2 and 24-h incubation with standard deviations (**b**). Asterisks designate the statistically significant difference (*p* < 0.05) compared to the highest value in the group

Recombinant bioluminescent *E. coli* increasing the bioluminescence in response to bioavailable Cu ions was applied to determine the role of internalized Cu ions in the antibacterial potency of Cu compounds (Fig. 2a). In parallel, chemical analysis was done to reveal dissolution of CuO (Fig. 2b). In the sub-toxic region, Cu compounds acted quite similar on sensor bacteria by increasing the bioluminescence of *E. coli* biosensor in parallel to the increase of the concentration of copper. There was, however, one exception: CuO−NH_4_^+^ NPs showed toxic properties (decline of luminescence) already at remarkably low concentrations (starting from 5 mg Cu/l), thus demonstrating the antibacterial effect independent of dissolved Cu ions (Fig. 2a). For other NPs, biosensor response was a function of Cu content and NP dissolution, being lowest for CuO (that had the lowest 0.5-h dissolution, 40%, Fig. 2b).

Thus, Cu biosensor suggested that Cu compounds exhibited antibacterial effects through bioavailable ionic Cu with the exception of CuO−NH_4_^+^ NPs. In contrast, CuO−NH_4_^+^ NPs were different from all the other Cu compounds by killing bacteria at remarkably low concentrations, even before the bioluminescence of biosensor was induced by Cu. Thus, we hypothesized that CuO−NH_4_^+^ exhibits specific partly Cu-independent antibacterial mechanism and studied the toxicity mechanisms of CuO−NH_4_^+^ and other NPs in more detail.

#### Ability of Cu compounds to induce ROS and inflammation

**Fig. 3:**
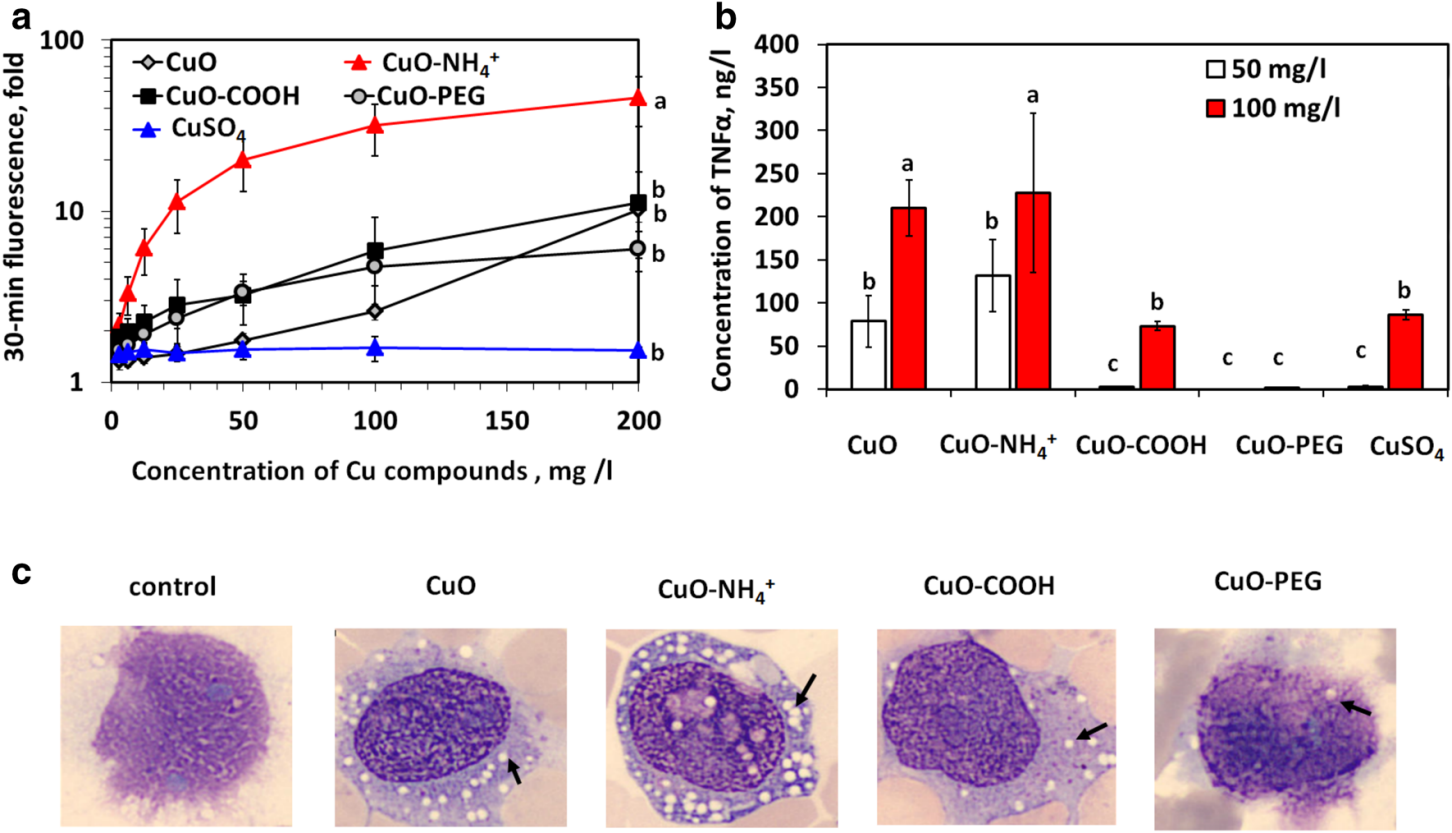
Oxidative and inflammatory potential of Cu compounds. Fluorescence of H_2_DCFA reflecting the ability of Cu compounds to produce reactive oxygen species in abiotic conditions in DI water (**a**). Concentrations of TNF-α in the supernatants of differentiated THP-1 cells exposed to Cu compounds in cell culture medium for 24 h (**b**) and representative light microscopy pictures of differentiated THP-1 cells exposed to equitoxic compound-based concentrations (24-h EC_20_) of Cu compounds for 24 h (**c**). Data presented as bars with the same letters are not statistically significant (*p* > 0.05) according to ANOVA analysis, whereas data presented as bars with different letters are statistically significant (*p* < 0.05). Arrows indicate localisation of the vacuoles

The results of the assay measuring abiotic ROS indicated that CuO−NH_4_^+^ NPs were very potent inducers of ROS (46-fold induction at concentration 200 mg/l), whereas other NPs were relatively poor ROS inducers (6- to 11-fold). CuSO_4_ did not induce ROS at any tested concentration (Fig. 3a). In addition, ELISA test revealed that CuO–NH_4_^+^ and also CuO NPs were the most potent inducers of TNF-α in differentiated THP-1 cells inducing TNF-α already at 50 mg/l, whereas CuO-COOH and CuSO_4_ induced TNF-α production starting from 100 mg/l (Fig. 3b) and CuO−PEG starting from 400 mg/l (data not shown). In general, TNF-α production correlated well with the EC_50_ numbers (Fig. 1a) and can be therefore considered as a marker of the cell death (Fink and Cookson 2005).

Using light microscopy, we also noticed extensive vacuolization in the cells exposed to CuO and especially to CuO−NH_4_^+^ NPs (Fig. 3c). Vacuolization has previously been suggested as a sign of inflammation and cell death (Shubin et al. 2016) and may indicate a distinct mechanism of toxicity of CuO−NH_4_^+^ NPs also in mammalian cells (macrophages) in vitro.

#### Measurement of cell-associated Cu from Cu compounds

**Fig. 4:**
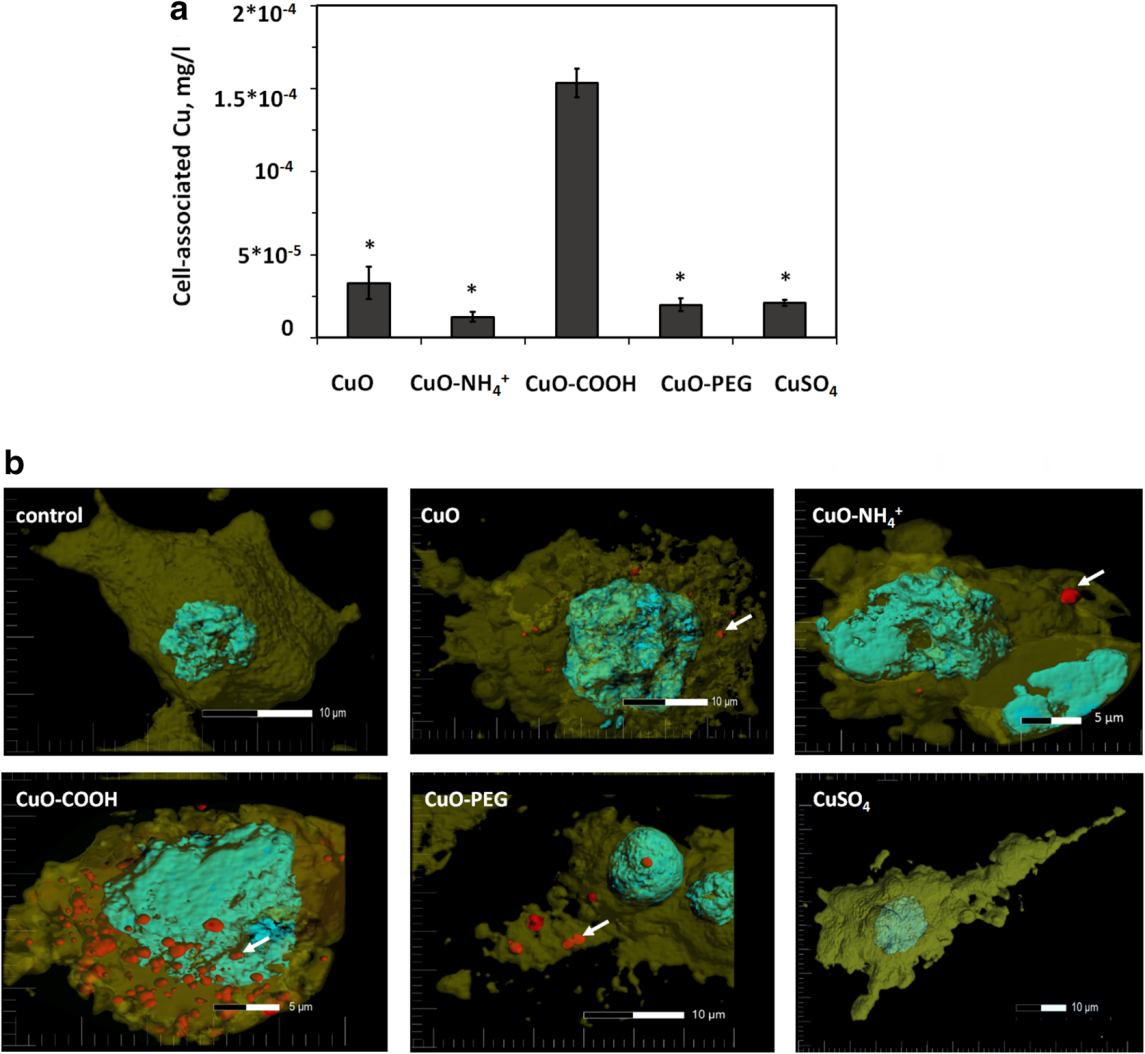
Interaction of Cu compounds with THP-1 cells. Concentration of copper associated with differentiated THP-1 cells after 24-h exposure to the equitoxic (EC_20_) concentrations of CuO NPs and CuSO_4_ (**a**). Asterisks show the statistically different values (*p* < 0.001). Representative confocal microscopy images of differentiated THP-1 macrophages exposed to equitoxic concentrations (24-h EC_20_) of Cu compounds for 24 h (**b**). Cell membranes were stained with Cell Mask Orange (yellow) and cell nucleus with DAPI (blue). Nanoparticles were visualized in red using reflective mode of the microscope. The arrows indicate the cellular localisation of the CuO NPs (color figure online)

To reveal the mechanisms of toxicity of Cu compounds, differentiated THP-1 cells and HACAT cells were exposed to equitoxic (24-h EC_20_) concentrations of Cu compounds for 24 h, washed and analyzed for Cu content. We assumed that Cu content mostly referred to intracellular Cu, but it cannot be excluded that some fraction of CuO NPs or dissolved Cu was tightly bound to cell surface and also detected by our analysis. Thus, the measured fraction was designated as “cell-associated Cu” combining intracellular Cu and CuO NPs and possible cell surface-bound Cu. We hypothezised that (i) if the toxicity of Cu compounds was caused solely by Cu ions, the amount of cell-associated Cu in the cells exposed to equitoxic concentrations of Cu compounds would be equal; (ii) if the toxicity was caused by additional factors (as suggested for CuO−NH_4_^+^), the amount of cell-associated Cu would be lower compared to the other CuO NPs and CuSO_4_. The experiment proved the latter option: there was significantly less Cu in both differentiated THP-1 and HACAT cells exposed to CuO−NH_4_^+^, than in case of other exposures (Fig. 4a and Fig. S4). Surprisingly, we observed about five- and eightfold higher amounts of cell-associated Cu in case of CuO−COOH compared to other NPs in both HACAT and differentiated THP-1 cells, respectively, suggesting that both cell lines had exceptional capacity to tolerate cell-associated Cu in the form of CuO−COOH. Therefore, we conducted the confocal microscopy study to confirm this result and visualize the cellular localization of CuO NPs.

#### Cellular localization of Cu compounds in mammalian cells

Differentiated THP-1 cells were exposed to equitoxic (24-h EC_20_) concentrations of Cu compounds for 24 h as in previous experiments and visualized with confocal microscopy (Figs. 4b, S5). The reflective mode of the microscope was optimized to visualize CuO NPs. However, some reflectance was seen also in a control group (shown in Fig. S5) that might be the dense inclusions of early lysosomes typical for macrophages (Douglas and Tuluc 2010). By combining Z-stacks into three-dimensional image, we observed that significantly more of CuO−COOH NPs were associated with cells compared to other NPs (Fig. 4b). Furthermore, confocal microscopy images indicated that most of the CuO−COOH NPs localized inside the cells (Supplementary video 1). Thus, the tolerance of THP-1 macrophages to internal Cu was exceptionally high for CuO−COOH NPs and low for CuO−NH_4_^+^ NPs. Interestingly, a similar phenomenon was previously shown for differently functionalized polystyrene NPs: the uptake of COOH-functionalized NPs by human monocyte-derived macrophages as well THP-1 monocytes was significantly higher than the uptake of polystyrene–NH_2_ NPs (Lunov et al. 2011a), but despite that polystyrene–NH_2_ NPs were toxic to the macrophages after 72-h exposure, while polystyrene–COOH NPs were not toxic (Lunov et al. 2011b).

### Multivariate analysis for CuO

**Fig. 5:**
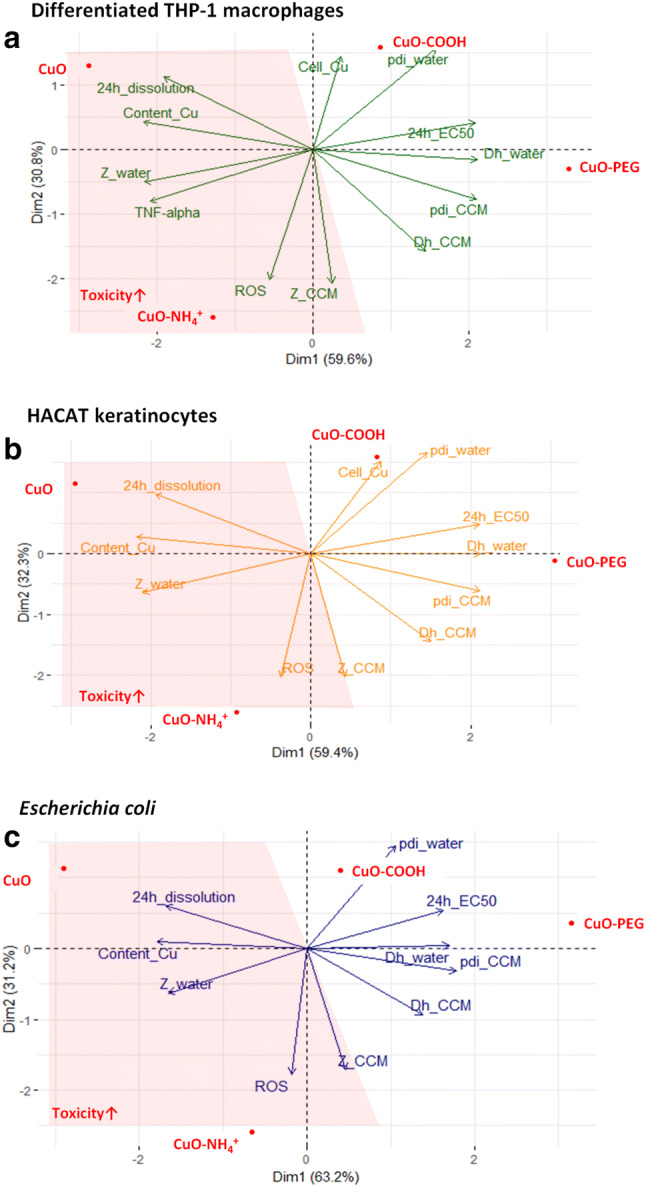
Properties contributing to toxicity of CuO compounds. Multivariate analysis of properties contributing to the variability of the toxicity of CuO, CuO–NH_4_^+^, CuO–COOH and CuO–PEG NPs to differentiated THP-1 macrophages (**a**), *E. coli* (**b**) and HACAT keratinocytes (**c**). *Z_water* surface charge in distilled (DI) water, *Z_CCM* surface charge in cell culture medium, *Cell_Cu* cell-associated Cu, *Dh_water* hydrodynamic size in DI water, *Dh_CCM* hydrodynamic size in cell culture medium, *pdi_CCM* pdi in cell culture medium, *pdi_water* pdi in water, *ROS* abiotic reactive oxygen species, *Content_Cu* copper content. More toxic compounds are highlighted in the red area (color figure online)

Finally, multivariate analysis was performed to evaluate the variability of different properties of CuO NPs and, thus, to estimate their contribution to the net toxicity. For this, toxicity data (Fig. 1a) and physico-chemical characterization data (Table 1, Figs. 1, 3) were fitted into scores plot that comprises the eigenvectors. The principal component analysis (PCA) was applied resulting in NP positions according to their variability (Fig. 5).

Since arrows indicated the direction of the increase of the values, 24-h EC_50_ vector denoted the direction of lower toxicity (increasing EC_50_ value) that was the most characteristic for CuO−COOH and CuO−PEG (Fig. 5a−c). To estimate the contribution of different physico-chemical parameters to NPs` toxicity, we focused on the properties localizing in the area as opposed to the EC_50_ value vector. Figure 5 shows that the properties contributing to increased toxic effects of unfunctionalized CuO and CuO−NH_4_^+^ NPs were more positive zeta-potential, higher Cu content, higher 24-h dissolution, ability to produce more abiotic ROS and in case of THP-1 cells also higher production of TNF-α. Importantly, the localization and the direction of the eigenvectors on the plot were strictly similar in case of mammalian cells (Fig. 5a, b) and bacteria (Fig. 5c), suggesting that the toxicity of CuO NPs to different cell types is influenced by the same variables.

## Discussion

Antibacterial metal-based NPs such as Ag, ZnO and CuO are usually purposely designed to inhibit the undesired growth of bacteria and are widely applied in medical and commercial products. However, it was shown that the toxicity range of CuO and Ag NPs to bacterial and mammalian cells in vitro may overlap, indicating the potential hazard of these NPs to human cells (Greulich et al. 2012; Bondarenko et al. 2013). In our comprehensive review on the toxicity of Ag, ZnO and CuO NPs to different organisms, we showed that among all studied NPs, CuO NPs had clear “particle-specific” toxic effect, i.e., NPs were more toxic than the Cu ions (on basis of Cu concentration) (Bondarenko et al. 2013)*.* Among all organisms, this effect was only evident for mammalian cells in vitro and yeast cells.

That brought us to the current study: to test new surface functionalizations that would possibly render CuO NPs less toxic to mammalian cells than to bacterial cells. Despite wide commercial use and toxicity of CuO NPs, there were no attempts to identify the surface functionalizations of NPs that would decrease the cytotoxicity of NPs to human cells without compromising antibacterial functions.

In the current study, we compared the toxicity and revealed the mechanisms of toxicity of unfunctionalized CuO NPs, CuO−COOH, CuO−NH_4_^+^, CuO−PEG and CuSO_4_ to bacteria *Escherichia coli* and to human cells: HACAT keratinocytes and macrophages differentiated from THP-1 monocytes in vitro. Our main aim was to identify the NP surface functionalizations that would improve the safety profile of CuO NPs to mammalian cells in vitro, while retaining sufficient antibacterial activity. We showed that the effect of the surface functionalizations of CuO NPs on toxicity is different for bacteria and human cells. Namely, while the toxicity of ionic CuSO_4_ was nearly identical to bacterial and human cells, CuO−COOH and CuO−PEG were significantly more toxic to bacteria than to human cells in vitro. In contrast, CuO−NH_4_^+^ was more toxic to human cells than to bacterial cells.

The effects of positively vs negatively charged nanomaterials to mammalian cells in vitro was previously addressed using, e.g., polystyrene NPs and carbon nanotubes. For example, it was shown that polystyrene–NH_2_ induced toxicity, lysosomal leakage and inflammasome activation and IL-1β production in primary human monocyte-derived macrophages (Loos et al. 2014), while polystyrene–COOH NPs were not toxic to the macrophages (Lunov et al. 2011b). In another study, carbon nanotubes functionalized with negatively charged COOH and PEG groups decreased the production of pro-fibrogenic cytokines and growth factors in human cell lines BEAS-2B and THP-1 compared to carbon nanotubes functionalized with NH_2_ or PEI (Li et al. 2014). In a recent study, it was shown that pristine, carboxylated and methylaminated, but not PEGylated, NPs worsened the pulmonary effects of CuO NPs in allergic airway inflammation mice model (Ilves et al. 2019). All these results are in line with our findings, showing that NH_4_^+^ functionalization renders CuO NPs that are especially toxic to human cells. Interestingly, from the studied NPs, CuO−NH_4_^+^-functionalized NPs were also the most toxic to bacteria, indicating additional universal mechanisms of toxicity unrelated to the active NP uptake, lysosomal damage and inflammation (that are not existing in bacteria). Most probably, unspecific toxicity component of CuO−NH_4_^+^ was mediated via ROS (Fig. 3a) and binding to the cell surface (Fig. 4). Surprisingly, the most common unfunctionalized CuO NPs were almost as toxic as CuO−NH_4_^+^ NPs to human cells in vitro (Figs. 1, 5a,b), especially for HACAT cells (24-h EC_50_ = 21.7 mg/l, the lowest toxicity value obtained in this study). It was previously demonstrated that 24-h EC_50_ of unfunctionalized CuO NPs to HACAT was around 30 mg/l (MTT reduction assay), and that CuO induced ROS, oxidative stress, DNA damage and apoptosis in HACAT cells (Alarifi et al. 2013). In our study, CuO induced ROS tenfold in abiotic conditions, but at irrelevantly high concentration (200 mg/l, Fig. 3a). Furthermore, CuO−NH_4_^+^ induced significantly more ROS, but was less toxic to HACAT than unfunctionalized CuO, suggesting that ROS-related mechanism was most likely not the primary mechanism of toxicity of unfunctionalized CuO to HACAT.

Our multivariate analysis of the properties contributing to the toxicity of Cu compounds revealed very similar patterns for mammalian cells in vitro and bacteria (Fig. 5). Zeta-potential, Cu content, dissolution and ability to induce ROS were the most significant parameters defining toxicity to all cell types, suggesting that the toxicity mechanisms of CuO NPs to bacterial and mammalian cells are largely similar. Thus, in addition to the surface functionalization (that can modulate the specificity of CuO NPs to some extent as shown in this study), attention should be paid to the specific targeting of bacterial cells using bacterial cell wall components-binding peptides, antibiotics or their combinations with NPs to achieve more specificity and enlarged therapeutic window.

Summarizing, CuO−COOH and CuO−PEG NPs can be considered as promising antibacterials to be used in biomedical applications, since they were significantly more toxic to bacteria than to human cells in vitro. It is well known that functionalization of NPs with PEG prevents the adsorption of proteins and, thus, the uptake of NPs by macrophages (Nguyen and Lee 2017) that most likely explains the reduced toxicity of CuO−PEG NPs to mammalian cells in our study. The reason why mammalian cells were able to tolerate high intracellular concentration of CuO−COOH NPs remains to be addressed. We speculate that COOH functionalization guides the NPs to the specific receptors and non-inflammogenic pathway, since it is known that NP interactions with the cell receptors impact their cellular localization, inflammatory properties and toxicity (Dobrovolskaia and McNeil 2007; Xia et al. 2008).

## Conclusions

Here, we report the benefits of the surface functionalization of CuO with carboxyl- or polyethylene glycol compared to unfunctionalized and ammonium-functionalized CuO NPs. Specifically, we showed that CuO−NH_4_^+^ NPs were significantly more toxic to human cells in vitro than to *E. coli* cells, probably because of their ability to induce inflammation (TNF-α) in human cells and ROS. The best therapeutic window was observed for CuO−COOH and CuO−PEG that can be recommended as antimicrobials.

Summarizing, we showed that the antibacterial potency vs safety profile of CuO NPs can be tuned with the surface functionalizations, and the effect of the surface functionalizations is different for bacteria and human cells. This knowledge can be used for the synthesis of more efficient and safer antimicrobials.

## Electronic supplementary material

Below is the link to the electronic supplementary material.

Supplementary file1 (PDF 1382 kb)

Supplementary file2 (AVI 19368 kb)
