# Supplementary material for: Surface carboxylation or PEGylation decreases CuO nanoparticles’ cytotoxicity to human cells in vitro without compromising their antibacterial properties
Source: Arch Toxicol. 2020 Apr 7;94(5):1561–73. doi: 10.1007/s00204-020-02720-7 (PMC7261733; doi:10.1007/s00204-020-02720-7)
Supplement: Supplementary file 1 — Supplementary file1 (PDF 1382 kb) [file 204_2020_2720_MOESM1_ESM.pdf]

SUPPLEMENTARY INFORMATION for

# Surface carboxylation or PEGylation decreases CuO nanoparticles' cytotoxicity to human cells *in vitro* without compromising their antibacterial properties


Anna-Liisa Kubo[1], Grigory Vasiliev[1,2], Heiki Vija[1], Jekaterina Krishtal[2], Vello Tõugu[2], Meeri Visnapuu[3], Vambola Kisand[3], Anne Kahru[1,4*], Olesja M. Bondarenko[1*]

[1] *Laboratory of Environmental Toxicology, National Institute of Chemical Physics and Biophysics, Akadeemia tee 23, Tallinn, Estonia.*

[2] *Department of Chemistry and Biotechnology, School of Science, TalTech, Akadeemia tee 15, Tallinn, Estonia.*

[3] *University of Tartu, Institute of Physics, W. Ostwaldi 1, Tartu, Estonia.*

[4] *Estonian Academy of Sciences, Kohtu 6, Tallinn, Estonia.*

*Corresponding authors

Contact: Tel./Fax: +372 6398382, E-mail: olesja.bondarenko@kbfi.ee; anne.kahru@kbfi.ee

**Table S1.** Details on testing conditions for Cu compounds.

| Test | Method | Exposure media for Cu compounds | Time (h) | 95% humidity and 5% $CO_2$ | Temperature (°C) |
|---|---|---|---|---|---|
| ***Tests with human cells*** | | | | | |
| **HACAT viability** | Alamar Blue assay | DMEM[#], 4.5 g/l glycose, L-glutamine, sodium pyruvate, 10% FBS[‡], 1% PEST | 24 | yes | 37 |
| **dTHP-1 viability** | Alamar Blue assay | CCM[Δ] (RPMI-1640[¤], 100 mM sodium pyruvate, 10% FBS, 1% PEST) | 24 | yes | 37 |
| **Cell-associated Cu, HACAT** | TXRF [¥] | DMEM, 4.5 g/l glycose, L-glutamine, sodium pyruvate, 10% FBS, 1% PEST | 24 | yes | 37 |
| **Cell-associated Cu, dTHP-1** | TXRF | CCM | 24 | yes | 37 |
| **NP cellular localization, dTHP-1** | Microscopy | CCM | 24 | yes | 37 |
| **TNF-α production, dTHP-1** | ELISA[£] | CCM | 24 | yes | 37 |
| ***Tests with bacterial cells*** | | | | | |
| ***E. coli* viability** | Alamar Blue assay | CCM without PEST | 24 | no | 37 |
| **Bioavailability of Cu to *E. coli*** | Cu ion biosensor *E. coli* [±] | CCM without PEST | 2 | no | 37 |
| ***Tests in abiotic conditions*** | | | | | |
| **Abiotic ROS** | $H_2$DCF fluorescence | DI-water[↑] | 0.5 | no | RT[illegible] |
| **Abiotic dissolution** | TXRF | CCM | 0.5, 2, 24 | yes | 37 |

¥ - Cu quantification with total reflection X-ray fluorescence analysis, £ - Enzyme-Linked Immunosorbent Assay, ± - *E. coli* MC1061 (pSLcueR/pDNPcopAlux), ‡-Fetal bovine serum, [illegible] - 10000 U/ml Penicillin and 10000 µg/ml Streptomycin, #-Dulbecco`s modified Eagle`s medium, ¤-Roswell Park Memorial Institute medium with L-glutamine, ↑- endotoxin free bi-distilled water, Δ- complete cell culture medium, [illegible]- room temperature

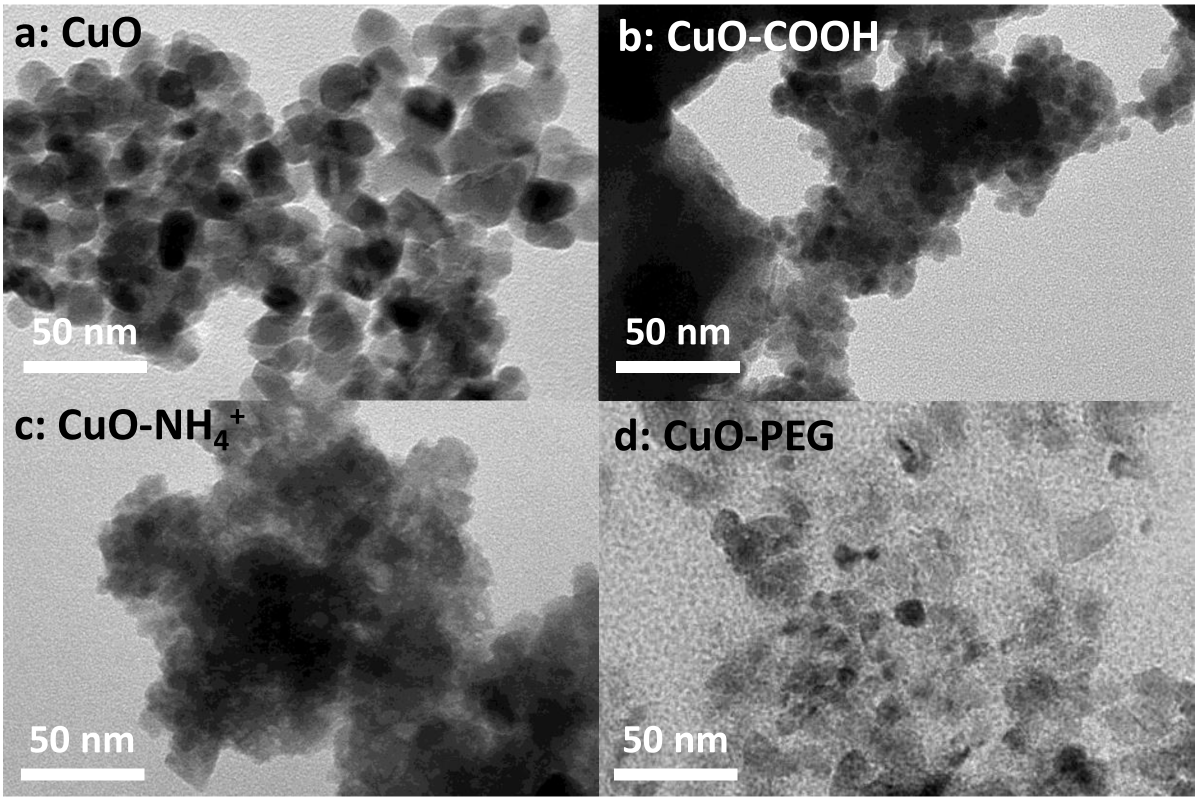


**Fig. S1.** Transmission electron microscopy (TEM) images of the studied nanoparticles: pristine CuO (a), carboxyl-functionalized CuO (CuO-COOH, b) ammonium-functionalized CuO (CuO-$NH_4^+$, c) and polyethylene glycol- functionalized CuO (CuO-PEG, d).

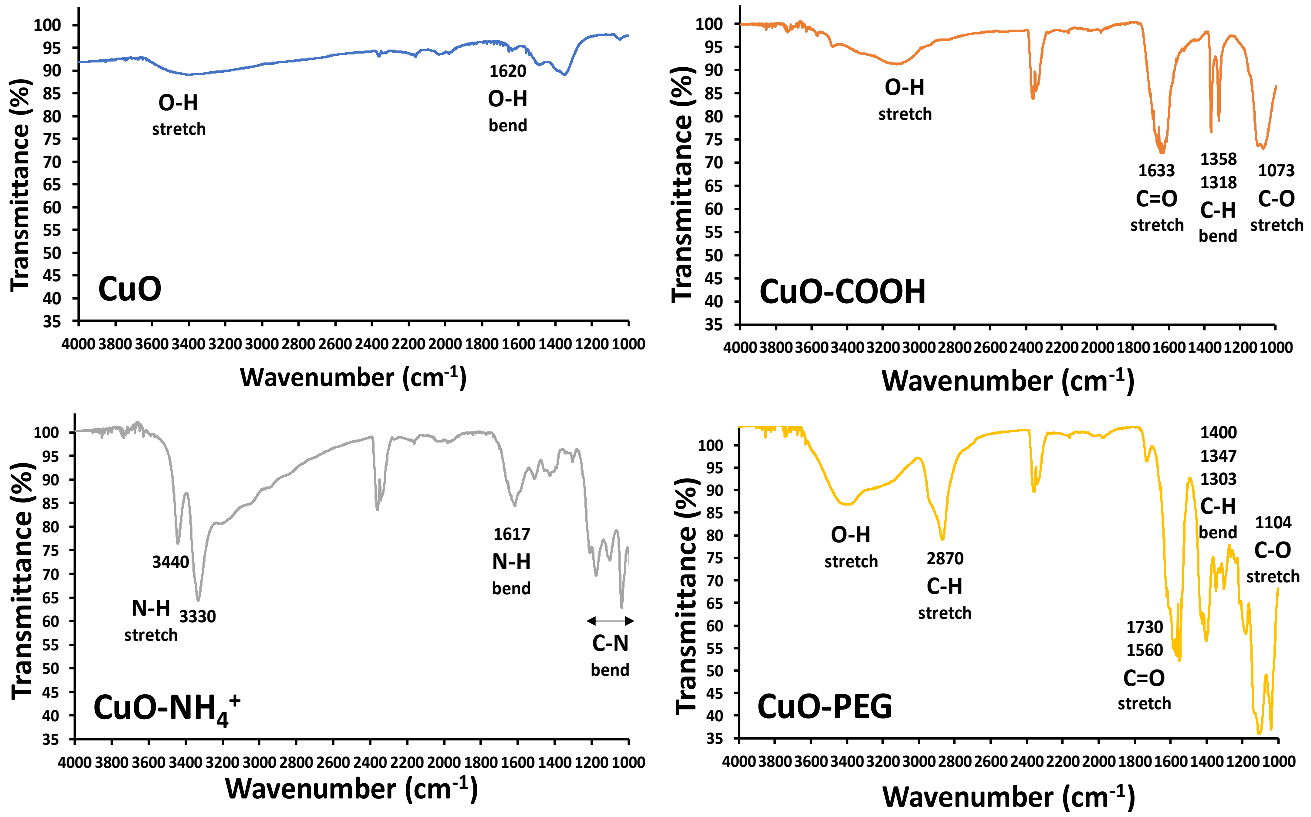


**Figure S2.** Fourier Transform Infrared Spectroscopy (FTIR) spectra of the studied nanoparticles: pristine CuO, carboxyl-functionalized CuO (CuO-COOH) ammonium-functionalized CuO ($CuO-NH_4^+$) and polyethylene glycol- functionalized CuO (CuO-PEG).

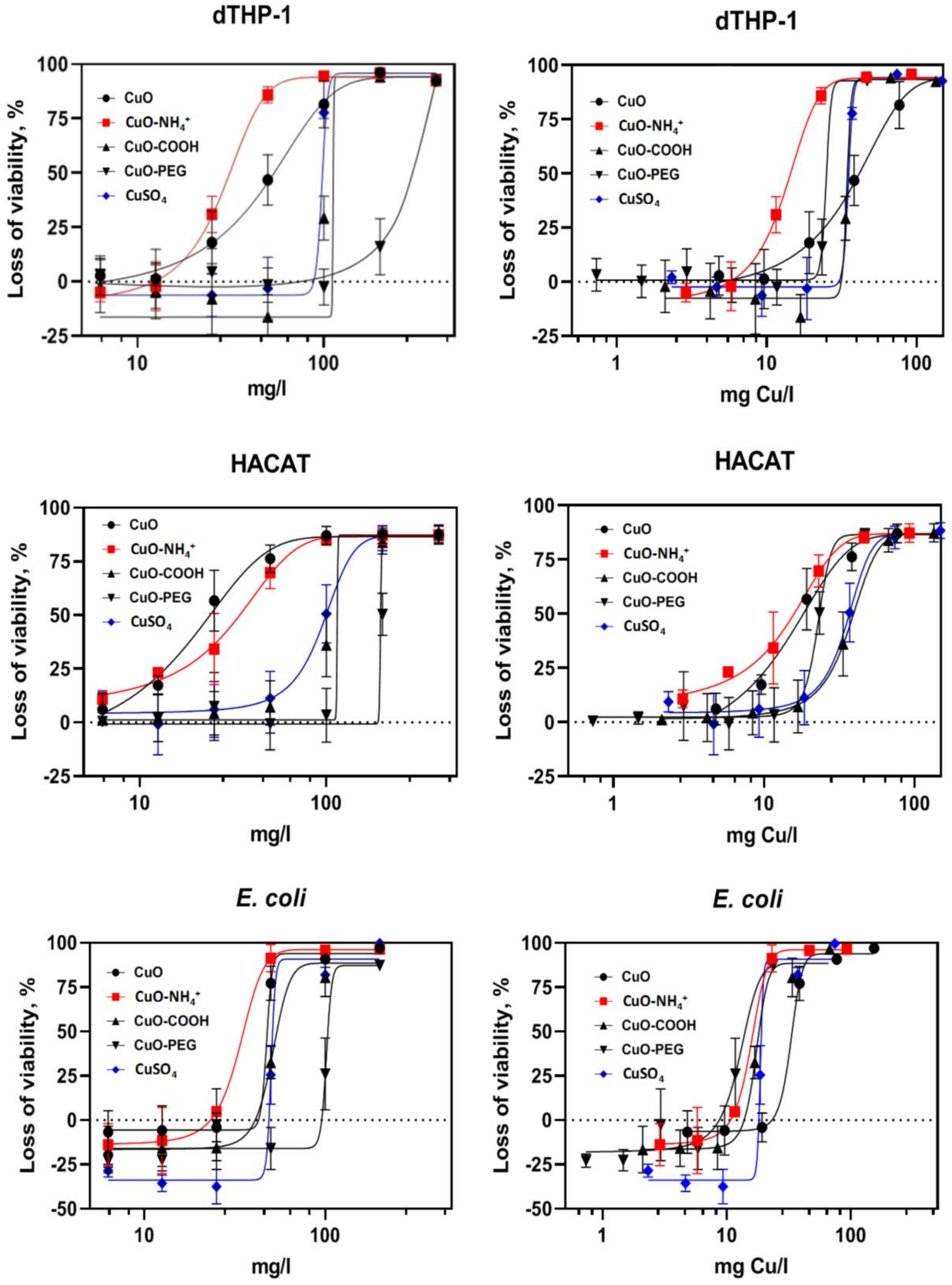


**Fig. S3.** Loss of viability of differentiated THP-1, HACAT keratinocytes and *Escherichia coli* after 24-h incubation with CuO NMs in RPMI1640 medium supplemented with 10% fetal bovine serum. The dose response curves were fitted using the GraphPad software based either on the compound (mg/l) or on copper (mg Cu/l).

.

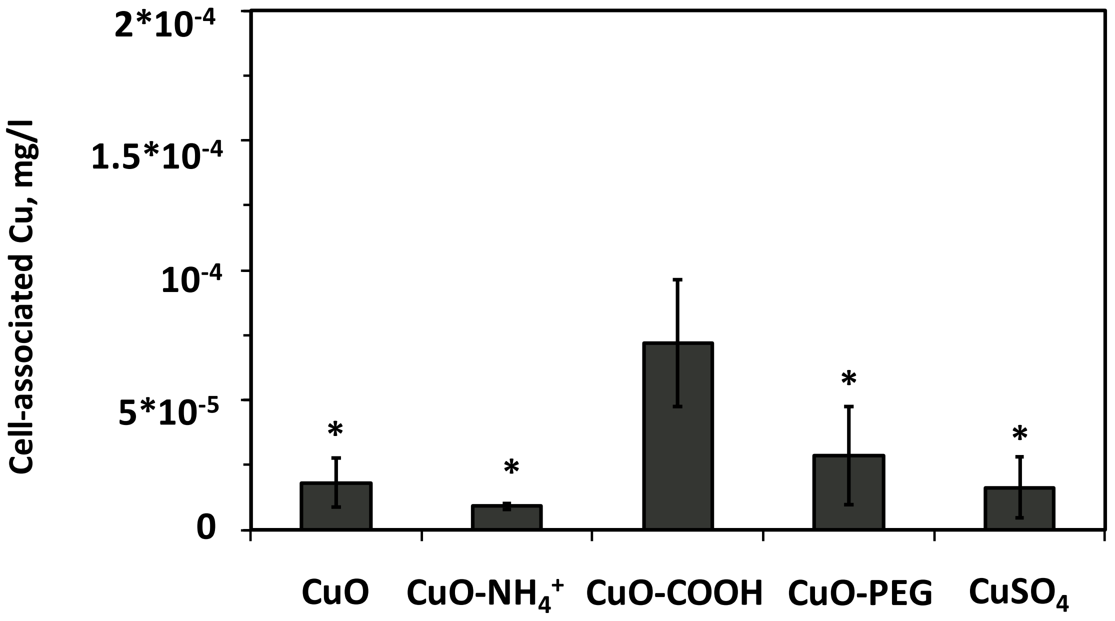


**Fig. S4.** Concentration of copper associated with HACAT cells after their 24-h exposure to the equitoxic ($EC_{20}$) concentrations of CuO NPs and $CuSO_4$. Cell-associated Cu was determined by TXRF (more information in the materials and methods section). The whiskers show the standard deviations and the asterisks designate the statistically significant difference ($p<0.05$) compared to the highest value according to the ANOVA analysis.

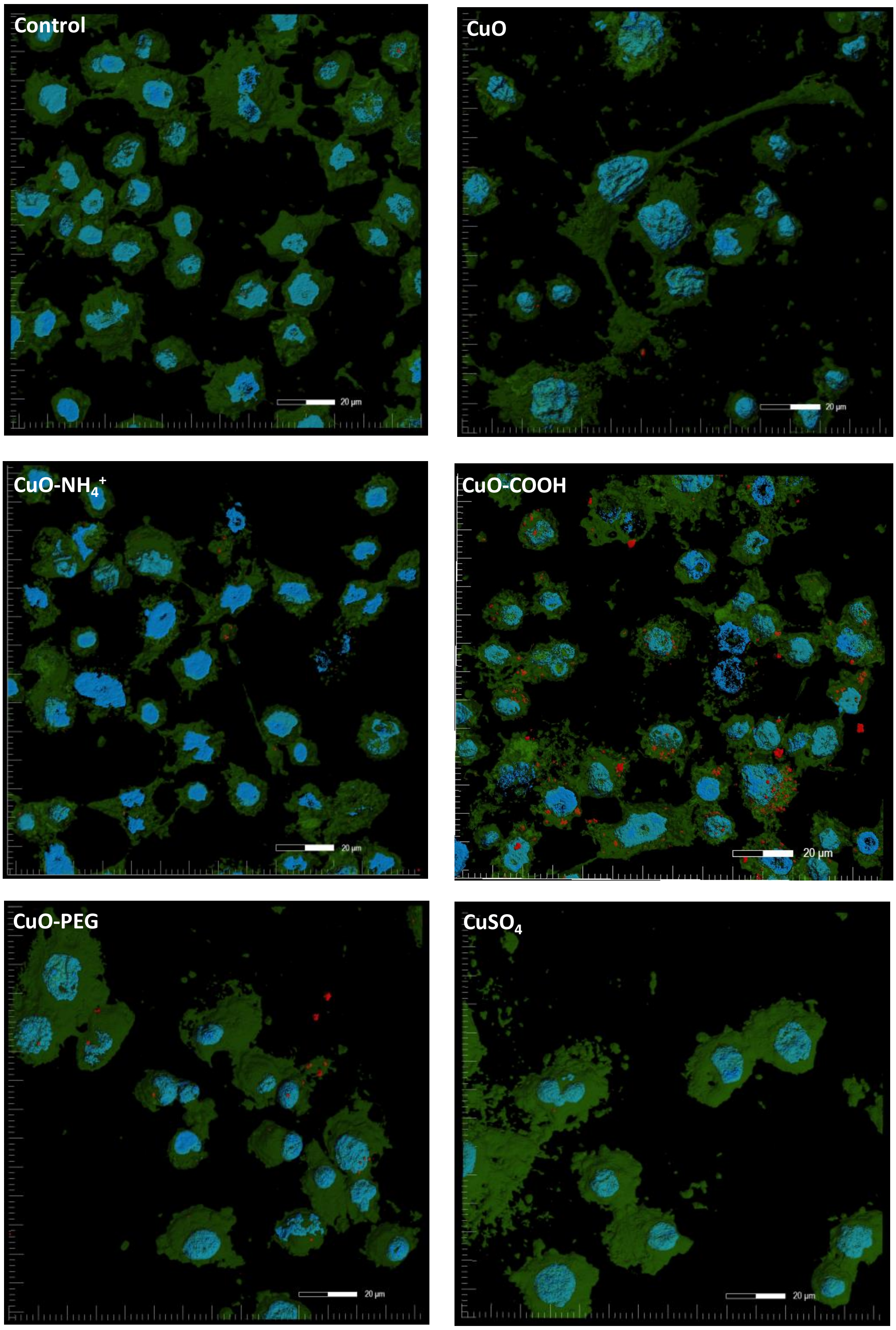


**Fig. S5.** Representative confocal microscopy figures of differentiated THP-1 macrophages exposed to Cu compounds (24-h $EC_{20}$: 27.3 mg/l for CuO NPs, 22.2 mg/l for CuO-$NH_2$, 90.6 mg/l for CuO-COOH, 211.4 mg/l for CuO-PEG and 85.4 mg/l for $CuSO_4$) for 24 h. Cell membranes were stained with Cell Mask Orange (yellow) and DAPI (blue). Nanoparticles were visualized in red using reflective mode of the microscope
